# Prenatal diagnosis of hypoplastic left heart syndrome: current knowledge

**DOI:** 10.1590/0100-3984.2023.0073

**Published:** 2023

**Authors:** Nathalie Jeanne Bravo-Valenzuela, Edward Araujo Júnior

**Affiliations:** 1 Department of Pediatrics, Pediatric Cardiology, School of Medicine, Universidade Federal do Rio de Janeiro (UFRJ), Rio de Janeiro, RJ, Brazil; 2 Department of Obstetrics, Escola Paulista de Medicina da Universidade Federal de São Paulo (EPM-Unifesp), São Paulo, SP, Brazil

**Keywords:** Prenatal diagnosis, Echocardiography, Prenatal screening, Hypoplastic left heart syndrome, Diagnóstico pré-natal, Ecocardiografia, Triagem pré-natal, Síndrome do coração esquerdo hipoplásico

## Abstract

Hypoplastic left heart syndrome (HLHS) is characterized by underdevelopment of the left-sided heart structures. The prenatal diagnosis of this congenital heart disease is crucial because a newborn with undiagnosed HLHS often presents with clinical signs of low cardiac output once the ductus arteriosus begins to close. With that in mind, the aim of this article was to perform a non-systematic review focusing on the key ultrasound features that can be used in the prenatal diagnosis of HLHS. Severe forms of HLHS are characterized by a markedly abnormal four-chamber view of the fetal heart (small left atrium, hypoplastic left ventricle, or abnormal mitral valve). The left ventricular outflow tract view allows the degree of hypoplasia in the tract to be evaluated and the diameter of the ascending aorta to be measured. The Z-scores are intended to aid in the diagnosis and follow-up of HLHS. In mild forms of HLHS, a right ventricle/left ventricle length ratio > 1.28 was the strongest predictor of a univentricular outcome.

## Abstract (pt)

A síndrome do coração esquerdo hipoplásico (SCEH) é caracterizada pelo subdesenvolvimento das estruturas cardíacas do lado esquerdo. O diagnóstico pré-natal dessa cardiopatia congênita é crucial, uma vez que recém-nascido com SCEH não diagnosticado apresenta, frequentemente, sinais clínicos de baixo débito cardíaco, quando o canal arterial começa a se fechar. Por isso, o objetivo deste artigo foi realizar uma revisão não sistemática sobre as principais características ultrassonográficas que podem ser usadas no diagnóstico pré-natal da SCEH. As formas graves de SCEH são caracterizadas por plano de quatro câmaras marcadamente anormal (átrio esquerdo pequeno, ventrículo esquerdo hipoplásico, válvula mitral anormal) do coração fetal. A visualização da via de saída do ventrículo esquerdo permite a avaliação do grau de hipoplasia dessa via e a mensuração da aorta ascendente. Os escores Z têm como objetivo auxiliar no diagnóstico e acompanhamento da SCEH. Nas formas leves da SCEH, a relação comprimento do ventrículo direito/comprimento do ventrículo esquerdo > 1,28 foi a variável mais forte para identificar o desfecho univentricular.

## INTRODUCTION

Hypoplastic left heart syndrome (HLHS) accounts for 1.4-3.8% of all cases of congenital heart disease (CHD) and is responsible for 23% of cardiac deaths occurring in the first week of life. It is characterized by underdevelopment of the left-sided cardiac structures. Prenatal diagnosis of HLHS is critical because a neonate with undiagnosed HLHS often presents with clinical signs of low cardiac output as the ductus arteriosus begins to close, with compromised systemic perfusion^(^1^)^. In addition, a restrictive foramen ovale (FO) accompanied by HLHS will require surgical intervention to open the interatrial septum *in utero* or immediately after birth. In this scenario, prenatal diagnosis is even more critical. Therefore, in this study, we review the ultrasound features relevant to the prenatal diagnosis of HLHS.

## HLHS - TERMINOLOGY AND MORPHOLOGY

The term HLHS describes a spectrum of cardiac malformations characterized by underdevelopment of the left side of the heart with severe obstruction of the left ventricular inflow and outflow tracts. In this context, it is important to note that the main morphological features of this CHD are hypoplasia of the left ventricle (LV) and its outflow tract^(^2^)^. In addition, the International Nomenclature Society has specified that HLHS encompasses a spectrum of CHD in which LV underdevelopment is associated with normally aligned great arteries without a common atrioventricular junction. Such features may help us distinguish HLHS from functionally univentricular hearts, which are defined as “a spectrum of congenital cardiac malformations in which the ventricular mass does not readily lend itself to partitioning that commits one ventricular pump to the systemic circulation, and another to the pulmonary circulation”^(^3^)^.

As previously described, it is well established that hearts with discordant atrioventricular or ventriculoarterial connections or with double outflow tracts or a common atrioventricular valve should not be included under the term HLHS. However, there is no consensus regarding whether the integrity or not of the interventricular septum should be considered one of the morphological features of HLHS^(^4^)^. In a recent review, Anderson et al.^(^2^)^ speculated that the likely allusion to the term syndrome grouping lesions with hypoplasia of the left heart was first described by Noonan et al.^(^5^)^. In their patients, the interventricular septum was categorized as either intact or not. However, when Lev^(^6^)^ reviewed the cases to be included in “hypoplasia of the aortic tract complex”, he decided to include those with an intact interventricular septum. Anderson et al.^(^2^)^ recommend that hearts with left ventricular hypoplasia should be divided as follows (Table 1): hearts with an intact ventricular septum; and hearts with a ventricular septal defect. The findings show that when the ventricular septum is intact, the hearts can be interpreted as having the characteristics of a disease acquired in fetal life.

**Table 1:** The main morphological features of HLHS.

Inclusion criteria	Exclusion criteria	Potential divergences
Hypoplasia of LV and its outflow tract Spectrum of CHD with underdevelopment of the LV and LVOT, together with normally aligned great arteries	Discordant atrioventricular or ventriculoarterial connections or double outflow tracts Common atrioventricular junction	Intact ventricular septum Ventricular septal defect

In addition to hypoplasia of the LV and aorta, the main anatomical features of HLHS are as follows: stenotic or atretic aortic and mitral valves; small or hypoplastic left atrium (LA); dilated right ventricle (RV, the dominant ventricle) with dilatation of the pulmonary artery trunk; and LV fibroelastosis in the presence of significant aortic atresia or stenosis, due to intrauterine coronary underflow. In the classic form of HLHS, the hearts have severe hypoplastic LV and atresia of the mitral and aortic valves.

Chromosomal abnormalities, such as Turner syndrome (45,X), trisomy 13, and trisomy 18, are seen in conjunction with HLHS in 4-5% of cases. In addition to genetic disorders such as Noonan syndrome, extracardiac anomalies are found in 10-25% of fetuses with HLHS^(^3^)^. The main associated malformations in HLHS are described in Table 2.

**Table 2:** The main associated anomalies in HLHS.

Cardiac anomalies	Genetic abnormalities
Shone’s complex^*^/Aortic coarctation	Chromosomal anomalies: 45,X;
Persistent of left superior vena cava	trisomy 13, and trisomy 18 Nonchromosomal abnormality: VACTERL association

* Consisting of left-sided obstructive lesions.

VACTERL, Vertebral defects, Anal atresia, Cardiac defects, Tracheo-Esophageal fistula, Renal anomalies, and Limb abnormalities.

## METHODS

For this non-systematic review, a search strategy was developed to identify articles published in English in the PubMed/PMC databases between 2018 and 2023. The following MeSH terms were used: “prenatal diagnosis”, “cardiac ultrasound screening”, “fetal echocardiography”, and “hypoplastic left heart syndrome”. Using the search terms above, we selected a total of 36 articles based on their titles and abstracts. Case reports and duplicate studies were excluded, as were studies on epidemiology or fetal cardiac interventions in HLHS, or even on the prenatal detection rate of cardiac ultrasound screening, as well as all studies in which the aim was not the prenatal ultrasound diagnosis of HLHS. On the basis of the inclusion and exclusion criteria, six full-text articles were selected for review. After the selected studies and their references had been reviewed, one study was added, making a total of seven studies.

## PRENATAL DIAGNOSIS OF HLHS BY ULTRASOUND/ECHOCARDIOGRAPHY

**Figure 1:**
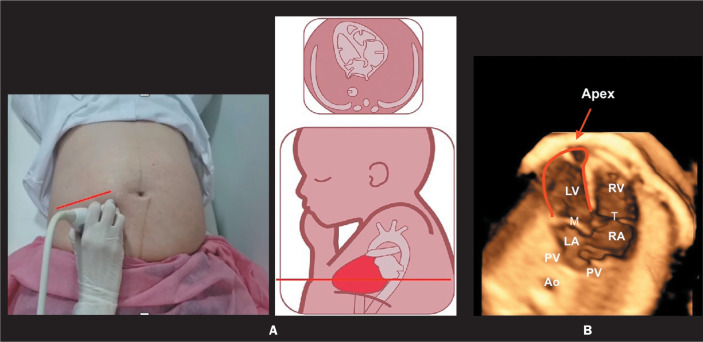
Cardiac ultrasound/echocardiography imaging showing how to scan the four-chamber view. Find the transaxial view of the fetal chest, perpendicular to the long axis of the fetus (A), and obtain a four-chamber view of the fetal heart (B). Note that, in the normal fetal heart depicted here, there is no discrepancy in the size of the ventricles and the LV forms the apex of the heart. M, mitral valve; T, tricuspid valve; RA, right atrium; Ao, aorta.

Fetal cardiac screening guidelines and training programs can maximize CHD detection rates by adding evaluation of the ventricular outflow tracts and upper mediastinal views (three-vessel and three-vessel trachea views) to the four-chamber view for cardiac sonography screening^(^7^-^9^)^. Despite the improvements in cardiac screening, major cardiac defects such as LV hypoplasia in HLHS are easily identified in the four-chamber view (Figure 1).

Fetuses with classic forms of HLHS (aortic and mitral atresia) have an abnormal four-chamber view with no inflow into the LV (mitral atresia). The ventricular chamber discrepancy due to the hypoplastic LV is the clue to suspect HLHS in the four-chamber view of the fetal heart. A small, thickened mitral valve, or even a muscular “bar” in mitral atresia, can be seen in the four-chamber view of the fetal heart. The absence of anterograde flow through the mitral valve on color Doppler confirms the diagnosis. Another important clue is that in these hearts the LV does not form the apex as in hearts with normal anatomy.

**Figure 2:**
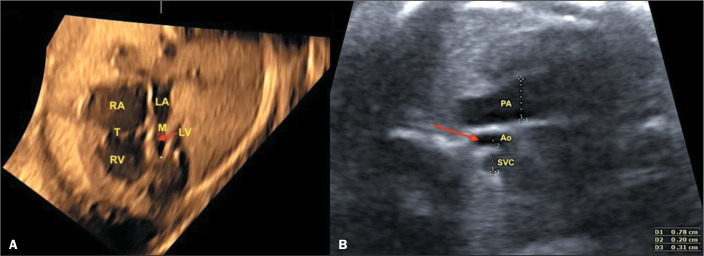
Fetal echocardiographic finding in a case of hypoplastic left heart syndrome. In the four-chamber view (A), note the small LA, hypoplastic LV with left-right chamber discrepancy and small mitral valve (arrow). In the three-vessel trachea view (B), note the discrepancy between the great arteries due to small aorta (arrow). Ao, aorta; M, mitral valve; RA, right atrium; SVC, superior vena cava; T, tricuspid valve.

In cases of aortic atresia and mitral stenosis, the LV is more recognizable and the reduced size of the LA can vary depending on the form of HLHS (severe, moderate, or mild). The view of the left ventricular outflow tract (LVOT) can confirm the diagnosis of HLHS by providing an evaluation of the degree of LVOT hypoplasia, making it possible to measure the ascending aorta, aortic isthmus, and descending aorta in millimeters and express them as Z-scores. The three-vessel and three-vessel trachea views allow the measurement of the ascending aorta and aortic isthmus. In cases of HLHS with aortic atresia, there is no anterograde flow across the aortic valve and a reverse flow in the aortic arch is detectable by color Doppler in the three-vessel and aortic arch views. In fact, in the upper mediastinum view, an increased pulmonary artery trunk/aorta ratio is seen due to the small aorta^(^10^)^, as illustrated in Figure 2.

**Figure 3:**
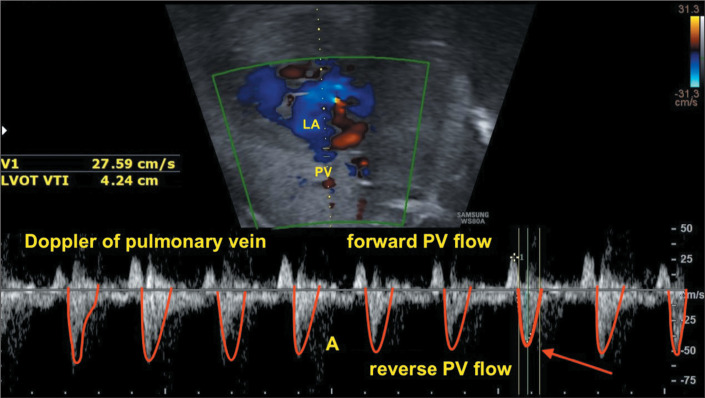
Doppler of the PV in a case of HLHS with restrictive FO. Doppler pattern showing a reversed A wave with a velocity > 40 cm/s. A, atrial wave of the PV.

Despite advances in prenatal diagnosis and therapeutic surgical management, the coexistence of FO restriction with HLHS is still a serious problem because it is associated with high mortality. Fetuses with restrictive interatrial shunts may be candidates for percutaneous enlargement of the patent FO *in utero* or immediately after delivery. Analysis of the pulmonary venous Doppler pattern is a useful tool in the evaluation of FO restriction. Pulmonary vein (PV) flow with an reversed A wave, with a velocity of up to 41 cm/s, a duration of more than 88 ms, or both, should raise attention to the diagnosis of a restricted FO. In addition to these PV Doppler parameters, a low ratio of forward PV flow to reverse flow, expressed as a velocity-time integral < 3, is a good predictor of neonatal instability and postnatal emergent intervention^(^11^-^13^)^, as depicted in Figure 3.

Prenatal assessment of RV function is important because the RV will be the dominant ventricle in the postnatal circulation after surgical intervention. On Doppler ultrasound, RV inflow should be assessed by determining the peak velocities of the E and A waves and, if available, by applying the E/E′ ratio; that is, tissue Doppler imaging^(^14^)^.

## RECENT DEVELOPMENTS AND OPEN QUESTIONS

In a review of the literature, Bravo-Valenzuela et al.^(^10^)^ described important features to improve the prenatal diagnosis of CHD by using ultrasound. The authors described the main features of HLHS and provided images with abnormal four-chamber views of the fetal heart due to hypoplastic LV, abnormal mitral valve and RV enlargement. In fact, the PV Doppler pattern with a reversed A wave is useful for identifying an interatrial shunt in HLHS.

Recently, Ximenes et al.^(^15^)^ conducted a study focusing on first-trimester ultrasound cardiac features that may allow early diagnosis of several major CHDs, including HLHS. The authors provided first-trimester ultrasound images with four-chamber views of the upper mediastinum and ventricular outflow tracts of fetuses with severe forms of HLHS, highlighting the discrepancy between the right and left cavities and between the great arteries. A small LA, hypoplastic LV, and small aorta are suggestive of a diagnosis of HLHS.

Edwards et al.^(^16^)^ described the measurements of cardiac structures in a cohort of fetuses with suspected left-sided cardiac lesions, in which 39 fetuses had HLHS. The authors described a smaller mitral valve, shorter LV length, smaller aortic valve, lower ascending aorta Z-scores, and a higher aorta/pulmonary artery ratio, as well as higher tricuspid valve-to-mitral valve ratio and RV-to-LV length ratio, as important cardiac measurements for predicting HLHS and a univentricular outcome. Among the described measurements, RV/LV length ratio > 1.28 was the strongest variable for identifying univentricular outcome. Similarly, in cases of borderline HLHS, Haberer et al.^(^17^)^ found that bidirectional or left-to-right FO flow, LV length Z-score < -2.4, and mitral valve length Z-score < 4.5 were predictors of a univentricular outcome in 80% of cases.

In a study conducted in China, Wu et al.^(^18^)^ showed that the mean Z-scores for LV length, LA diameter, and ascending aorta diameter were significantly lower (≤ 3.5) in a cohort of 79 fetuses with HLHS than in controls. The authors emphasized that the evaluation of cardiovascular Z-score equations should benefit the diagnosis and follow-up of HLHS cases.

Regarding FO restriction and HLHS, Sokolowski et al.^(^13^)^ described prenatal diagnosis of FO restriction as a predictor of long-term hospitalization, with a low positive predictive value for an urgent Rashkind procedure. Jadczak et al.^(^12^)^, in a study of HLHS associated with FO restriction, observed higher short-term mortality in cases with earlier development and longer presence of FO restriction, regardless of the degree of restriction. Those authors found that, although the PV forward/reverse flow velocity-time integral ratio is a good predictor of the need for intervention, it does not influence survival rates. In fact, they described the presence of ultrasound signs of fetal infection as a potential risk factor for FO restriction in fetuses with HLHS.

## CONCLUSION

The discrepancy between the right and left cavities in the four-chamber view on cardiac ultrasound/echocardiography should alert to the diagnosis of HLHS. Severe forms of HLHS are characterized by a markedly abnormal four-chamber view of the fetal heart (small LA, hypoplastic LV, abnormal mitral valve). When the LVOT view is used, a hypoplastic aorta, hypoplastic LV, and small LA support the diagnosis of HLHS. In addition, the outflow tract view allows the assessment of the degree of LVOT hypoplasia to be assessed by measuring the ascending aorta. The Z-scores are intended to aid in the diagnosis and follow-up of HLHS. In mild forms of HLHS, a RV/LV length ratio > 1.28 appears to be the strongest variable for identifying a univentricular outcome. Earlier FO restriction is associated with higher mortality in HLHS, regardless of the degree of restriction. Assessment of the PV forward/reverse flow ratio by Doppler is useful for predicting the need for emergent intervention related to FO restriction but does not influence survival rates in HLHS. There is a need for further studies of the ultrasound parameters that can be used as predictors of mortality, and new lines of research should be developed.
